# Dual and recombinant infections: an integral part of the HIV-1 epidemic in Brazil.

**DOI:** 10.3201/eid0501.990108

**Published:** 1999

**Authors:** A Ramos, A Tanuri, M Schechter, M A Rayfield, D J Hu, M C Cabral, C I Bandea, J Baggs, D Pieniazek

**Affiliations:** Centers for Disease Control and Prevention, Atlanta, Georgia, USA.

## Abstract

We systematically evaluated multiple and recombinant infections in an HIV-infected population selected for vaccine trials. Seventy-nine HIV-1 infected persons in a clinical cohort study in Rio de Janeiro, Brazil, were evaluated for 1 year. A combination of molecular screening assays and DNA sequencing showed 3 dual infections (3.8%), 6 recombinant infections (7.6%), and 70 (88.6%) infections involving single viral subtypes. In the three dual infections, we identified HIV-1 subtypes F and B, F and D, and B and D; in contrast, the single and recombinant infections involved only HIV-1 subtypes B and F. The recombinants had five distinct B/F mosaic patterns: Bgag-p17/Bgag-p24/Fpol/Benv, Fgag-p17/Bgag-p24/Fpol/Fenv, Bgag-p17/B-Fgag-p24/Fpol/Fenv, Bgag-p17/B-Fgag-p24/Fpol/Benv, and Fgag-p17/B-Fgag-p24/Fpol/Fenv. No association was found between dual or recombinant infections and demographic or clinical variables. These findings indicate that dual and recombinant infections are emerging as an integral part of the HIV/AIDS epidemic in Brazil and emphasize the heterogenous character of epidemics emerging in countries where multiple viral subtypes coexist.


### Dual and Recombinant Infections: An Integral Part of the HIV-1 Epidemic in Brazil

**Artur Ramos,*† Amilcar Tanuri,† Mauro Schechter,† Mark A. Rayfield,* Dale J. Hu,* Maulori C. Cabral,† Claudiu I. Bandea,* James Baggs,‡ and Danuta Pieniazek***

*Centers for Disease Control and Prevention, Atlanta, Georgia, USA;
†Universidade Federal do Rio de Janeiro, Brazil;
‡Emory University, Atlanta, Georgia, USA

We systematically evaluated multiple and recombinant infections in an HIV-infected population selected for vaccine trials. Seventy-nine HIV-1 infected persons in a clinical cohort study in Rio de Janeiro, Brazil, were evaluated for 1 year. A combination of molecular screening assays and DNA sequencing showed 3 dual infections (3.8%), 6 recombinant infections (7.6%), and 70 (88.6%) infections involving single viral subtypes. In the three dual infections, we identified HIV-1 subtypes F and B, F and D, and B and D; in contrast, the single and recombinant infections involved only HIV-1 subtypes B and F. The recombinants had five distinct B/F mosaic patterns: $B^{gag\text{-}p17}/B^{gag\text{-}p24}/F^{pol}/B^{env}$, $F^{gag\text{-}p17}/B^{gag\text{-}p24}/F^{pol}/F^{env}$, $B^{gag\text{-}p17}/B\text{-}F^{gag\text{-}p24}/F^{pol}/F^{env}$, $B^{gag\text{-}p17}/B\text{-}F^{gag\text{-}p24}/F^{pol}/B^{env}$, and $F^{gag\text{-}p17}/B\text{-}F^{gag\text{-}p24}/F^{pol}/F^{env}$. No association was found between dual or recombinant infections and demographic or clinical variables. These findings indicate that dual and recombinant infections are emerging as an integral part of the HIV/AIDS epidemic in Brazil and emphasize the heterogenous character of epidemics emerging in countries where multiple viral subtypes coexist.

Our understanding of the global molecular epidemiology of HIV-1 infections has improved substantially in recent years. Remarkable antigenic diversity, especially in the viral envelope, has emerged among HIV isolates worldwide (1). As the HIV/AIDS pandemic grows, viral strains are becoming more geographically dispersed, and the simultaneous presence of multiple subtypes in a given region is now common (2). Mixed infections and recombinants involving sequences of distinct HIV-1 subtypes (mosaics) are being recognized, but their prevalence and effect on the pandemic have not been fully evaluated (3). Consequently, the distribution of dual infections and mosaic viruses among different populations and the changes of this distribution over time are still relatively unknown. In addition, scientists are increasingly interested in possible differences in the transmission, epidemiologic patterns, and natural history of HIV-1 infections caused by more than one viral subtype and recombinant genomes. Finally, the efficacy of HIV-1 vaccines, primarily developed against subtype B viruses, may differ against more divergent recombinant variants and mixed infections of distinct HIV-1 subtypes. Such knowledge is vital to understanding the relevant role of mixed infections as a prerequisite for recombination and could be applied immediately in molecular epidemiology and immunotherapy.

Studies using convenience samples first documented mixed infections caused by viruses of subtypes B and E in Thailand (4). Subsequently, such studies have identified cases of dual infections with subtypes B, F, C, and D in Brazil (5-7), B and F in Puerto Rico (8), A and C in Rwanda (9), and triple HIV-1 infection with groups O and M of different clades in a single Cameroonian AIDS patient (10). In addition, potential dual infections have been detected by molecular screening assays among HIV-1 infected populations in Uganda and Kenya, where subtypes A and D coexist (11). The consequences of HIV-1 mixed infections may profoundly influence the

Address for correspondence: Danuta Pieniazek, HIV/Retrovirus Diseases Branch, Division of AIDS, STD, and TB Laboratory Research, CDC, 1600 Clifton Road, MS G19, Atlanta, GA, 30333, USA; fax: 404-639-1010; e-mail: dxp1@cdc.gov.

dynamic of the pandemic through altered patterns of viral transmission and pathogenesis. Moreover, the resulting genetic variation may lead to the emergence of new HIV variants, including those with altered antigenicity and reduced sensitivity to detection by current diagnostic assays. The global emergence of such variants is exemplified in the impact of two HIV-1 recombinants of subtypes A/E and G/A on epidemics in Thailand and in certain parts of Central Africa, respectively (1,12-14). Interestingly, the presumptive parental subtypes E and G have not been identified.

Because Brazil has been selected as a World Health Organization field site for HIV-1 vaccine evaluation programs, priority has been given to extensive molecular examination of the prevalence and genetic diversity of HIV-1 strains circulating in the country. By November 1997, 116,277 AIDS cases had been reported to the Brazilian AIDS Control Program of the Ministry of Health (15). The 293 HIV-1 strains that have been molecularly characterized document that, although four HIV-1 subtypes—B, F, C, and D—are circulating in Brazil, only subtypes B and F are common (16-21). Moreover, five HIV-1 dual infections were identified in 21 HIV-infected patients during testing of molecular techniques that would discriminate between HIV-1 infections caused by single and multiple subtypes (5-7). Also, two cases of B/F recombinants have been found accidentally through sequence analysis of the *env* region (22). These data only indicate the potential for dual and recombinant infections in Brazil, where multiple subtypes circulate; they cannot, however, be used to assess the frequency of these infections among the HIV-infected population. In this study, we evaluated the proportion of HIV-1 dual and recombinant infections among 79 patients enrolled in a prospective clinical cohort study in Rio de Janeiro (an area where HIV-1 subtypes B and F are common).

### The Study

### Population

Part of an ongoing prospective clinical cohort study established in 1991 by the AIDS program of the Federal University of Rio de Janeiro, the HIV-1-infected patients have been continuously enrolled in the cohort for consultation, treatment, evaluation of different clinical parameters during the progression of the disease, and assessment of the genetic variation of HIV-1 strains (23). With informed consent, blood samples were obtained from the 79 patients, who consecutively attended the clinic between October and December 1994. For the purposes of this study, samples collected in 1994 were compared as needed, and follow-up specimens were collected 1 year later. The clinical profile of patients was based on the medical evaluation at the time of blood collection. Because the patients were randomly selected, the findings presented in this article likely reflect the trends in the HIV/AIDS epidemic in the Rio de Janeiro area. Epi Info, Version 6 program (CDC, Atlanta, GA, USA) was used to calculate frequencies, means, and analyses of variance of demographic information, clinical stages, and laboratory parameters.

### Design

To investigate the proportion of mixed infections, we must distinguish between those involving two or more distinct HIV-1 strains and infection with a single intersubtype recombinant strain. By definition, the mixed infection occurs when multiple phylogenetically distinct copies of the same gene representing different viral genomes are present within one patient (24). In contrast, in the mosaic strain, different viral regions within the same genome are classified by phylogenetic analysis into different subtypes (3). Potential HIV-1 mixed infections involving distinct HIV-1 variants were segregated from single infections caused by only one subtype by using a restriction fragment length polymorphism (RFLP) screening assay of the *prt* gene (5,7). The restriction map profiles of the viral *prt* allow the segregation of HIV-1 strains to subtypes A, B, C, D, and F (Figure 1A). AluI digestion patterns separate subtypes A, C, and F from subtypes B and D. Sequential restriction analysis of *prt* using HinfI, BclI, and ScaI restriction enzymes further differentiates among these two subtype groups. The simultaneous occurrence of more than one digestion pattern indicates a potential mixed infection (Figure 1B). We selected recombinants among RFLP-subtyped single infections by additionally subtyping the C2-V3 *env* region with the heteroduplex mobility assay (25). Subtype discrepancies between *prt* and *env* regions were considered potential HIV-1 recombinants. All potential multiple infections

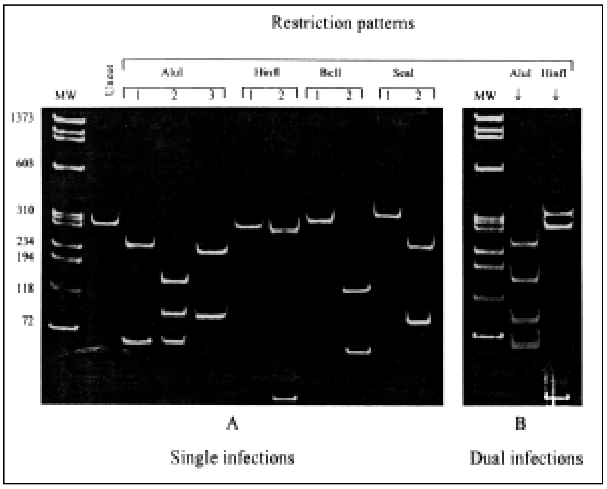

Figure 1. Differentiation between single (A) and dual (B) HIV-1 infections by the restriction fragment length polymorphism analysis of the polymerase chain reaction–amplified *prt*. A: Three AluI digestion patterns represent subtypes A, C, and F (pattern 1) and subtypes B and D (patterns 2 and 3); two HinfI patterns represent subtypes D (pattern 1) and B (pattern 2); two BclI patterns represent subtypes F (patterns 1) and A and C (pattern 2); two ScaI patterns represent subtypes A (pattern 1) and C (pattern 2). B: Two AluI digestion patterns (1 and 2) in the dually infected patient with HIV-1 subtypes F and B; two HinfI patterns (1 and 2) in the patient infected with subtypes B and D viruses.

and recombinants were analyzed by sequence analysis. The search for recombinants was further expanded by sequence analysis of the entire p17 *gag* or a 311-bp of the p24 *gag* fragment or both among preselected *prt-env* recombinants, all *prt-env* subtype F, and some *prt-env* subtype B viruses.

### Polymerase Chain Reaction (PCR)

Uncultured or cultured peripheral blood mononuclear cells from patients were used for nested-PCR of the entire HIV-1 protease gene (*prt*, 297-bp), p24 *gag* fragment (311-bp), and C2-V3 domain of *env* (565-bp) (6,7). The outer primers for amplification of a 717-bp gag fragment spanning the entire 17 *gag* region and a 311-bp of p24 *gag* were LTRF: 5'GGGCTAATTTGGTCAAAAAGAAG; nucleotide position: 6-28, HIV-$1_{MN}$, and P24RA: 5'ATGTCACTTCCCCTTGGTTCT; nucleotide position: 1482-1502. The inner primers were P17F: 5'GCAAGAGGCGAGGGGCAGCAGCCG; nucleotide position: 716-739, and P17R: 5'CCCATTCTGCAGCTTCATTGA; nucleotide position: 1413-1433. The outer primers for amplification of a 1,444-bp fragment from p24 *gag* to the reverse transcriptase region were P24F1: 5'ATAGAGGAAGAGCAAAACAAAA; nucleotide position: 1,099 to 1,120, and MOPR1: 5'AAAATTGGAGTATTGTATGGATT; nucleotide position: 2,724 to 2,746. The inner primers were P24FA: 5'CAAAATTACCCTATAGTGCA; nucleotide position: 1,177 to 1,196, and MOPR2: 5'GGTCCATCCATTCCTGGTTT; nucleotide position: 2,601 to 2,620. PCR conditions were the same for amplification of all viral regions (7).

### Sensitivity of Detection of Dual Infections

To determine the sensitivity of detection of dual infections by the RFLP assay, we mixed 5, 10, 25, 50, and 100 copies of subtypes B and F cloned proviral DNA in equal proportions or in ratios 1:2, 1:4, 1:10, and 1:20 for nested PCR amplification of *prt* (Table 1). For each

Table 1. Sensitivity of detection of HIV-1 dual infections caused by viruses of subtypes B and F

| No. of viral subtypes | | Ratio | No. of experi- | AluI digestion patterns of prt | | |
|---|---|---|---|---|---|---|
| F | B | F:B | ments | B | F | B&F |
| 100 | 100 | 1:1 | 1 | - | - | + |
| 50 | 50 | 1:1 | 4 | - | - | + |
| 25 | 25 | 1:1 | 4 | - | + (1) | + (3) |
| 10 | 10 | 1:1 | 4 | - | - | + |
| 5 | 5 | 1:1 | 4 | - | + (3) | + (1) |
| 100 | 5 | 20:1 | 2 | - | + (1) | + (1) |
| 100 | 10 | 10:1 | 2 | - | + (1) | + (1) |
| 100 | 25 | 4:1 | 2 | - | + | - |
| 100 | 50 | 2:1 | 2 | - | - | + |
| 5 | 100 | 1:20 | 1 | - | - | + |
| 10 | 100 | 1:10 | 1 | - | - | + |
| 25 | 100 | 1:4 | 1 | - | - | + |
| 50 | 100 | 1:2 | 1 | - | - | + |
| 100 | 0 | | 3 | - | + | - |
| 50 | 0 | | 3 | - | + | - |
| 25 | 0 | | 3 | - | + | - |
| 10 | 0 | | 3 | - | + | - |
| 5 | 0 | | 2 | - | + | - |
| 0 | 100 | | 3 | + | - | - |
| 0 | 50 | | 2 | + | - | - |
| 0 | 25 | | 3 | + | - | - |
| 0 | 10 | | 3 | + | - | - |
| 0 | 5 | | 3 | + | - | - |

Absence (-) and presence (+) of AluI digestion pattern of *prt* characteristic for subtype B, subtype F, and combination of subtypes B and F (Fig. 1). The cloned proviral DNA of HIV-1 subtypes B and F spanning a 1444-bp fragment from p24 *gag* to *rt* was used for the nested PCR amplification of *prt*. Amplified products were digested with AluI restriction enzyme, and the presence of two digestion patterns was analyzed on a 10% polyacrylamide gel by ethidium-bromide staining.

combination, one to four independent PCR reactions were run. The simultaneous amplification of the viral *prt* of subtypes B and F was evaluated by the RFLP assay. PCR controls included DNA templates of single HIV-1 subtypes.

### Cloning, Sequencing, and Phylogenetic Analysis

The PCR-amplified proviral *prt* sequences from potential dual infections were cloned by using the Original TA Cloning Kit (Invitrogen, Carlsbad, CA, USA). DNA from 30 clones of each specimen was screened for distinct HIV-1 sequences by the RFLP assay (7). Double-stranded viral DNA from selected clones or from direct PCR-amplified *prt*, p17 and p24 *gag*, and C2-V3 *env* products was cycle-sequenced in both directions with fluorescent dye–labeled sequencing terminators (26). Sequencing reactions were run in an automated DNA sequencer (Applied Biosystems, Foster City, CA, USA). The sequences were aligned by the CLUSTAL multiple sequence alignment program (27). After gaps were eliminated, the aligned sequences were analyzed by the maximum likelihood method, with the fastDNAml program, which uses randomized data input and global rearrangement (28). Additionally, the neighbor joining method (PHYLIP package version 3.5c [29]) was used, with or without bootstrapping. The stability of the tree's topology was tested by pruning (removing one sequence from the alignment and rerunning the phylogenetic analysis). The SIV-cpz sequences (GenBank accession no. X52154) were used as outgroups. HIV-1 sequences generated in this study have been submitted to GenBank.

### Results

### Detection of Mixed Infections

RFLP analysis of the *prt* gene showed the simultaneous presence of two different digestion patterns in specimens from 10 of 79 patients. A complex pattern composed of elements of AluI patterns #1 (subtypes A, C, or F) and #2 (subtypes B or D) was identified in nine patients, and a combination of two HinfI digestion patterns (subtypes B and D) was found in one patient (Figure 1B). These data suggest that each of 10 patients could be infected with multiple distinct HIV-1 subtypes. The remaining 69 samples were classified as single infections caused by viruses of *prt* subtypes B (59) and F (10).

The PCR-amplified viral *prt* products from these 10 patients were cloned and sequenced to confirm the presence of mixed infections. Sequence analysis showed the simultaneous presence of two distinct HIV-1 variants in only three patients: BR45, BR62, and BR83. The nucleotide divergence between the two *prt* sequences within the patients was 6.8% for BR45, 7.4% for BR62, and 7.1% for BR83, indicating two distinct HIV-1 variants in each of these patients (6). Phylogenetic analysis confirmed these findings and demonstrated that the divergent HIV-1 *prt* sequences segregated into subtypes B and F (BR45), subtypes D and F (BR62), and subtypes B and D (BR83) (Figure 2a). In the remaining seven specimens, the observed RFLP results were consequences of either point mutations in AluI restriction site (five cases) or G A hypermutation (two cases), which occurred across one of the sequences within each specimen and destroyed the defining AluI sites. These changes in AluI restriction sites gave rise to genetically distinct quasispecies within the patients but did not represent distinct subtypes, as further confirmed by phylogenetic analysis (e.g., BR55-1 and BR55-2, and BR99-1 and BR99-2 in Figure 2a). Thus, despite the presence of mixed AluI digestion patterns in these seven specimens, they were classified as single infections of subtype B variants.

To address the issue of potential laboratory contamination, we collected repeat blood samples from dually infected patients approximately 1 year later and processed them on separate occasions. (Blood was unlikely to be contaminated during collection because a disposable vacutainer system was used to obtain each blood sample.) The sequence data from the first and second blood samples of each person showed a 98% to 99% similarity. Also, the viral sequences from these patients were distinct from those of laboratory strains (Figure 2a; 855M, 8,986, and 9,001) commonly used as standards.

### Sensitivity of Detection of Dual Infections

To investigate the sensitivity of the RFLP screening method, we performed reconstruction experiments, in which two distinct viral DNA templates of subtypes B and F were analyzed in the same reaction mixture (Table 1). When equal proportions of 10 to 100 HIV-1 DNA template copies were used for *prt* amplification, two viral subtypes could be simultaneously detected in all

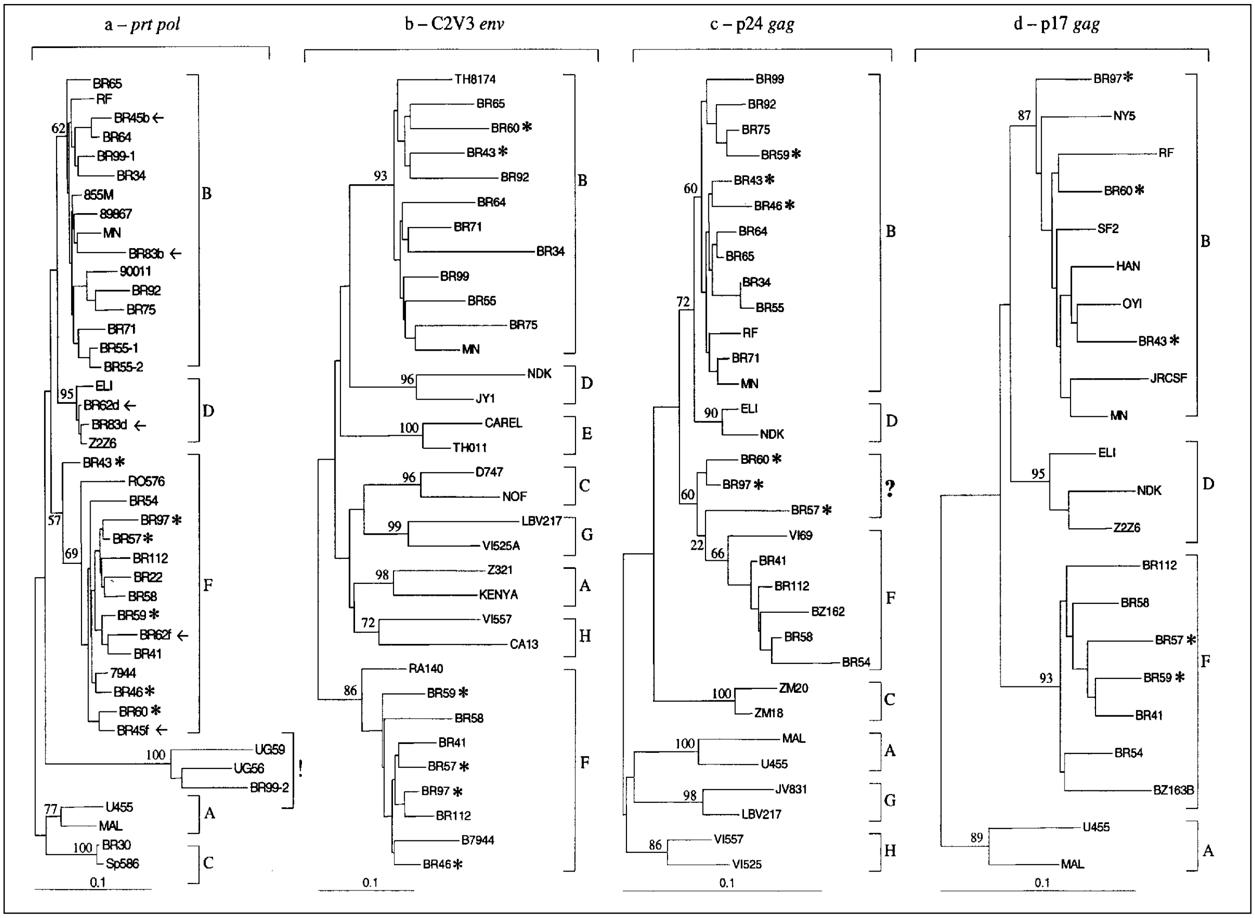

Figure 2. Phylogenetic classification of HIV-1 sequences from Brazilian patients (denoted with BR prefix). The trees were constructed on the basis of DNA sequences of *prt* (a), *env* (b), *gag*-p24 (c), and *gag*-p17 (d) by the neighbor-joining method. Numbers at the branch nodes connected with subtypes indicate bootstrap values. An arrow indicates dually infected specimens; an asterisk shows viral sequences, which clustered into different lineages depending on which parts of viral genome were analyzed; ! represents hypermutated sequences; ? represents unclassified subtype of p24 *gag* sequences. The distinct HIV-1 subtypes are delineated. The scale bar indicates an evolutionary distance of 0.10 nucleotides per position in the sequence. Vertical distances are for clarity only. GenBank accession numbers: *prt* [AF099155-99171;AF079986-79989;AF079991; and AF079994-79996]; *env* [AF113560-113576]; p17*gag* [AF115443-115451]; p24*gag* [AF115780-115797].

but one of 19 experiments). However, when five or fewer copies of each viral subtype were used, two subtypes were identified in only one of four assays; in the remaining three assays either subtype B or subtype F amplicons, but not both, were found. Similarly, in experiments containing varying proportions of subtypes B and F DNA templates, the simultaneous presence of two viral subtypes could be detected in only 8 (66%) of 12 experiments. In comparison, 5 to 100 copies of a single viral subtype (B or F) were routinely amplified and identified in all control reactions.

### Detection of Intersubtype HIV-1 Recombinants

Parallel RFLP/heteroduplex mobility assay screening for HIV-1 subtypes in the *prt* and *env* regions identified two potential recombinants among 76 single infections. Subtype F *prt* and subtype B *env* were found in both specimens BR43 and BR60 during this initial screening. The remaining specimens were classified into subtypes F (n = 8) and B (n = 66) in both *prt* and *env* regions. These *prt-env* potential recombinants, all subtype F specimens, and eight selected subtype B samples were further evaluated by sequence analysis, which confirmed the results of the screening assays (Figure 2a, 2b).

The search for the HIV-1 recombinant genome in these 18 samples was further expanded to the *gag* region. Mosaic sequences of subtypes B and F were found within the p17 or p24 *gag* (Table 2, Figure 2, discussion below) in four *prt-env* subtype F variants (BR46, BR57, BR59, and BR97). Similarly, in one of two *prt-env* recombinants (BR60), the *gag* sequences had a mosaic pattern. In contrast, the p24 *gag* sequences of eight *prt-env* subtype B specimens were homogeneous and also classified as subtype B (Figure 2). Taken together, the comparative molecular analysis of *gag*, *pol*, and *env* regions allowed the identification of six specimens that carried HIV-1 recombinant genomes, representing five distinct mosaic structures: $B^{gag\text{-}p17}/B^{gag\text{-}p24}/F^{pol}/B^{env}$, $F^{gag\text{-}p17}/B^{gag\text{-}p24}/F^{pol}/F^{env}$, $B^{gag\text{-}p17}/B\text{-}F^{gag\text{-}p24}/F^{pol}/F^{env}$, $B^{gag\text{-}p17}/B\text{-}F^{gag\text{-}p24}/F^{pol}/B^{env}$, and $F^{gag\text{-}p17}/B\text{-}F^{gag\text{-}p24}/F^{pol}/F^{env}$.

The potential crossover breakpoints within 717-bp of the p17-p24 *gag* mosaic sequences were examined by comparison with nucleotide signature patterns characteristic for subtypes B and F viruses (Figure 3). We performed comparative analyses with aligned DNA sequences of recombinants (BR57, BR59, BR60, and BR97), subtype B (MN and BR43), and subtype F variants (BR41, BR54, BR58, and BR112). This analysis confirmed an intragene recombination within p24 *gag* in specimens BR57, BR60, and BR97—a finding consistent with our failure to phylogenetically assign these *gag* sequences to any known subtype (Figure 2c).

The putative breakpoints within the intragene recombinant sequences were located between nucleotides 97 and 137 in sample BR57 and between nucleotides 173 and 213 in BR60 and BR97 (Figure 3). The exact breakpoint position could not be determined because of extensive sequence homology between subtypes B and F in this viral region. This analysis also revealed putative crossover breakpoints for variants BR57 and BR59 in proximity to the coding region for the p17-p24 protein-processing site. The potential breakpoints between the second half of *gag* and the beginning of *pol* region in variants BR43, BR46, and BR59 are being investigated.

To examine the possibility of in vitro recombination during PCR amplification (30), we performed PCR amplification of the long fragments covering the *gag* and *prt* area in the endpoint- diluted lysates (31). To ensure that endpoint PCR products were amplified from single copy templates, we used samples only from dilutions at which 1 of 10 PCR amplifications were productive for further sequence analysis. The comparative analysis of the entire p17 *gag*, p24 *gag*, and *prt* sequences demonstrated 98% homology between the undiluted and diluted lysates—strong evidence that the recombinant sequences were not a result of the PCR amplification process.

Table 2. p17/p24 *gag*, *prt*, and C2-V3 genetic subtyping of HIV-1 DNA sequences[a] from peripheral blood mononuclear cells collected from 18 patients in Rio de Janeiro

| | | Genotypes | | | |
|---|---|---|---|---|---|
| Specimen | No. | *gag* p17 | *gag* p24 | *prt* | C2-V3 |
| Group 1[b] | 8 | ND[c] | B | B | B |
| Group 2[d] | 4 | F | F | F | F |
| (BR46) | | (NA[e]) | (B) | (F) | (F) |
| (BR59) | | (F) | (B) | (F) | (F) |
| (BR57) | | (F) | (B/F) | (F) | (F) |
| (BR97) | | (B) | (B/F) | (F) | (F) |
| (BR60) | | (B) | (B/F) | (F) | (B) |
| (BR43) | | (B) | (B) | (F) | (B) |

[a]Recombinant specimens are shown in parentheses; B/F – mosaic structure within a 311-bp of the p24 *gag* fragment consisting of subtypes B and F sequences.
[b]Group 1: BR34, BR52, BR55, BR64, BR65, BR71, BR75, and BR92.
[c]ND=not done.
[d]Group 2: BR41, BR54, BR58, and BR112.
[e]NA = not available due to negative PCR.

### Epidemiologic and Clinical Characteristics

The mean ages of patients infected with HIV-1 of single subtype B or F were not different from those with dual or recombinant infections (p = 0.77) (Table 3). In addition, the patients did not differ significantly by gender, risk group, and clinical stage of disease (p = 0.44 to p = 0.48). The patients infected with HIV-1 subtype B (403) and subtype F (854) did, however, differ (p = 0.04) by mean CD4 counts. Although dates of seroconversion were not known for all patients, the earliest HIV-positive results were reported among patients infected with subtype B (in agreement with previous observations that the spread of subtype B viruses occurred earlier than other HIV-1 subtypes in Brazil [16,17]), which might explain the difference in CD4 counts.

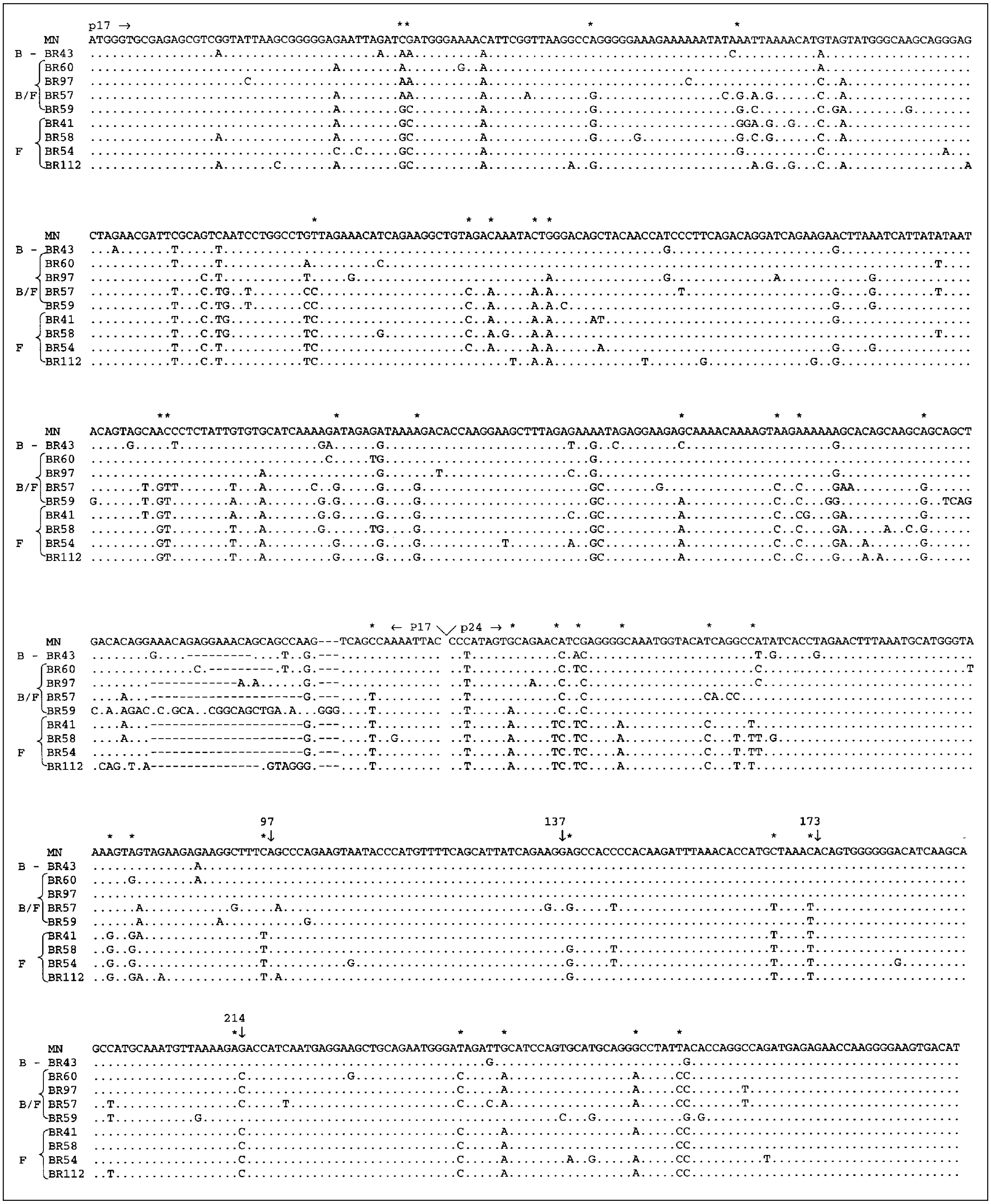

Figure 3. Analysis of the putative recombination within the *gag* region. The aligned sequences were classified into subtype B, subtype F, and recombinant subtype B/F on the basis of linearity of subtype assignment for the p17-p24 *gag* region. Asterisks show characteristic nucleotide patterns for subtypes B and F sequences; dots represent nucleotides homologous to the MN *gag* sequence; dashes indicate gaps introduced to maintain the alignment; and arrows indicate the potential recombination regions within the p24 *gag* fragment. The nucleotide position is marked.

Table 3. Characteristics of the study population

| Characteristic | Dual infection n=3 | Recombinant infection n=6 | Subtype B n=66 | Subtype F n=4 |
|---|---|---|---|---|
| Gender (Female/male) | 1/2 | 3/3 | 24/42 | 3/1 |
| Mean age in years (range) | 36 (30-44) | 34 (25-45) | 37 (23-60) | 34 (26-43) |
| Clinical stage[a] | | | | |
| 1 | 3 | 5 | 29 | 4 |
| 2 | - | 1 | 17 | - |
| 3 | - | - | 14 | - |
| 4 | - | - | 6 | - |
| Mean CD4 cells (range) | 527 (404-743) | 484 (190-821) | 403 (59-1281)[b] | 823 (570-1270) |
| Years of 1st serologic tests | | | | |
| 1985-1987 | - | - | 2 | - |
| 1988-1991 | 2 | 2 | 30 | 2 |
| 1992-1994 | 1 | 4 | 34 | 2 |
| Heterosexual | 2 | 5 | 27 | 4 |
| Homosexual | 1 | - | 19 | - |
| Bisexual | - | - | 8 | - |
| Blood transfusion recipient | - | - | 3 | - |
| Intravenous drug user | - | - | 1 | - |
| Multiple factors | - | 1 | 2 | - |
| Unknown | - | - | 6 | - |

[a]WHO staging system [32].
[b]Based on data available for 54 patients.

### Conclusions

HIV-1 infections caused by dual and intersubtype-recombinant genome may be relatively common among HIV-1-infected Brazilians. Using both heteroduplex mobility assay and RFLP screening methods, as well as sequencing, we identified three dual (3.8%) and six recombinant (7.6%) infections involving distinct viral subtypes among 79 HIV-1-infected persons from Rio de Janeiro.

We chose viral *prt* to screen for dual infections because this highly conserved region provides the best opportunity for simultaneous amplification of distinct HIV-1 variants in the same PCR reaction. Proviral *prt* sequences can be routinely amplified from approximately 95% of all analyzed seropositive samples collected from the Americas, Asia, Africa, and Europe (data not shown). Moreover, the RFLP assay of the *prt* gene is convenient for screening a large number of samples (5). The detection of B/F, B/D, and F/D dual infections is in agreement with our 1993 study among patients from the same Rio de Janeiro cohort, which showed five dual HIV-1 infections involving subtypes F and B (one case), F and D (one case), and B and C (three cases of familial clustering) among 21 HIV-infected persons (5-7). Interestingly, dual infections involving HIV-1 subtype D continue to be detected in patients from Rio de Janeiro, an area with a high predominance of HIV-1 subtypes B and F, but rare subtype D, single infections. Because retrospective specimens (peripheral blood mononuclear cells) were not available for these patients, we could not confirm whether the acquisition of mixed strains was sequential (superinfection) or simultaneous. However, combination of subtypes F or B with rare subtype D viruses among some dual infections may suggest that in these cases two viral strains might be acquired through cotransmission rather than through superinfection.

All naturally occurring HIV-1 dual infections are likely not detected by current methods. First, quantitative differences in two distinct viral DNA templates in the sample can lead to selective PCR amplification of only one subtype. Second, despite targeting conserved genes such as *prt*, some divergent viral strains may escape PCR amplification because of primer mismatches. Finally, a single nucleotide mutation in the endonuclease restriction site can abrogate the recognition pattern and distort detection of dual infections in the RFLP analysis. These observations suggest that the rate of HIV-1 mixed infections within this Brazilian cohort might be even higher than 4%. Our previous findings of five dual infections among 21 patients from the same cohort support this assumption. If we take into account these five cases, the percentage of mixed infections caused by viruses of distinct subtypes circulating between 1993 and 1994 among 100 patients analyzed from this Rio de Janeiro cohort would increase to 8%.

The potential underestimate of mixed infections is highlighted by the additional detection of 6 (7.6%) distinct recombinants within the cohort. This finding is consistent with the estimated 5% to 10% intersubtype mosaics among HIV-1 genomes in the Los Alamos database (3). Interestingly, all recombinant or mosaic genomes described in this report involved only subtypes B and F viral regions, although dual infections caused by other subtypes were also circulating in this cohort. Moreover, our results indicate that recombination between *gag* and *pol* (*prt*) regions is more frequent than between *pol* and *env* and lead to speculation that such stable B/F mosaics have selective advantage. Such observations support the assumption that recombination within the *gag* gene occurs more often than within other viral regions (33) and emphasize the need for rapid subtyping methods specific to the *gag* region.

To investigate the potential impact of dual and recombinant infections on the clinical status of patients, we compared the clinical and demographic characteristics of these patients with those of patients infected with one nonrecombinant viral subtype. Epidemiologic information for all 79 patients was available only at the first draw of blood. Although the results did not show significant differences between the two groups, the possibility of differences exists.

Our findings provide the first baseline measure of the range of HIV variability in Rio de Janeiro from 1993 to 1994 and the proportion of dual and recombinant infections among the HIV-infected Brazilian population. Future systematic molecular epidemiologic surveys of HIV heterogeneity in Rio de Janeiro may show the potential changes in the molecular profile of the HIV/AIDS epidemic over time; the laboratory tools we used to identify single, dual, and recombinant infections may be useful in such investigations. Nevertheless, our study on genetic variation of HIV-1 subtypes among blood donors from the state of Rio de Janeiro documented the presence of mosaic viruses of subtypes B and F and subtypes B and D in blood units collected in 1996 (34). Moreover, recent genetic analysis of viral strains collected in 1997 from HIV-1-infected patients living in Manaus (a city in Brazil's Amazon region) showed the presence of dual infections and recombinants caused by subtypes B and F viruses (A. Tanuri, pers. comm.). Therefore, these data indicate that the heterogenic pattern of HIV-1 infections, first observed in Rio de Janeiro, also exists in other regions of Brazil.

Our findings indicate that mixed infections and mosaic viruses may be more common in worldwide epidemics than previously thought and, therefore, may provide a basis for developing AIDS vaccines and predicting the global evolution of HIV.

### Acknowledgments

We thank Priscilla Swanson for her help in sequencing and Renu Lal for critical review of the manuscript.

Artur Ramos is a graduate fellow at the Federal University of Rio de Janeiro, Rio de Janeiro, Brazil. His laboratory research focuses on HIV-1 genetic variability worldwide. His research interests include application of molecular biology techniques to epidemiology of HIV.

### References

1. Leitner T. Genetic subtypes of HIV-1. In: Myers G, Foley B, Mellors JW, Korber B, Jeang KT, Wain-Hobson S, editors. Human Retroviruses and AIDS. Theoretical Biology and Biophysics, Los Alamos National Laboratory, Los Alamos, New Mexico; 1996: III28-40.
2. Hu DJ, Dondero TJ, Rayfield MA, George JR, Schochetman G, Jaffe HW, et al. The emerging genetic diversity of HIV. The importance of global surveillance for diagnostics, research, and prevention. JAMA 1996;275:210-6.
3. Robertson DL, Sharp PM, McCutchan FE, Hahn BH. Recombination in HIV-1. Nature 1995;374:124-6.
4. Artenstein AW, VanCott TC, Mascola JR, Carr JC, Hegerich PA, Gaywee J, et al. Dual infection with human immunodeficiency virus type 1 of distinct envelope subtypes in humans. J Infect Dis 1995;171:805-10.
5. Pieniazek D, Janini LM, Ramos A, Tanuri A, Schechter M, Peralta JM, et al. HIV-1 patients may harbor viruses of different phylogenetic subtypes: implications for the evolution of the HIV/AIDS pandemic. Emerg Infect Dis 1995;1:86-8.
6. Janini LM, Tanuri A, Schechter M, Peralta JM, Vicente AC, Dela Torre N, et al. Horizontal and vertical transmission of human immunodeficiency virus type 1 dual infections caused by viruses of subtypes B and C. J Infect Dis 1998;177:227-31.
7. Janini LM, Pieniazek D, Peralta JM, Schechter M, Tanuri A, Vicente AC, et al. Identification of single and dual infections with distinct subtypes of human immunodeficiency virus type 1 by using restriction fragment length polymorphism analysis. Virus Genes 1996;13:69-81.

8. Moran N, Soler A, Flores I, Alegria M, Vera M, Pieniazek D, et al. Multi-strain HIV-1 infection among female sex workers in Puerto Rico: emerging pattern of HIV-1 epidemic. 4th Conference on Retroviruses and Opportunistic Infections [abstract 170]. Washington DC, January 1997.
9. Kampinga G, Simonon A, van de Perre P, Karita E, Maellati P, Goudsmit J. Primary infections with HIV of women and their offspring in Rwanda: Findings of heterogeneity at seroconversion, coinfection and recombinants of HIV-1 subtypes A and C. Virology 1997;227:63-76.
10. Takehisa J, Zekeng L, Mlura T, Ido E, Yamashita M, Mboudjeka I, et al. Triple HIV-1 infection with group O and group M of different clades in a single Cameroonian AIDS patient. J Acquired Immune Defic Syndr 1997;14:81-2.
11. Pieniazek D, Janini ML, Ramos A, Bandea C, Soriano V, Downing R, et al. Mixed infections with HIV-1 strains of different phylogenetic subtypes: implication for the evolution of the HIV/AIDS pandemic. 3rd Conference on Retroviruses and Opportunistic Infections [abstract]. Washington DC, January 1996.
12. Carr JK, Salminen MO, Koch D, Gotte A, Artenstein AW, Hegerich PA, at al. Full length sequence and mosaic structure of a human immunodeficiency virus type 1 isolate from Thailand. J Virol 1996;70:5935-43.
13. Wasi C, Herring B, Raktham S, Vanichseni S, Mastro TD, Young NL, et al. Determination of HIV-1 subtypes in injecting drug users in Bangkok, Thailand, using peptide-binding enzyme immunoassay and heteroduplex mobility assay: evidence of increasing infection with HIV-1 subtype E. AIDS 1995;9:843-9.
14. Abimicu AG, Stern TL, Zwandor A. Subgroup G HIV-type 1 isolates from Nigeria. AIDS Res Hum Retroviruses 1994;10:1581-3.
15. Brazilian Ministry of Health. Boletim Epidemiologico-AIDS: Semana Epidemiologica-36 a 48-setembro a novembro de 1997. 1997;10:no 04 [http//www.aids.gov.br].
16. Potts KE, Kalish ML, Lott T, Orloff G, Luo CC, Bernard MA, et al. Genetic heterogeneity of the V3 region of the HIV-1 envelope glycoprotein in Brazil. AIDS 1993;7:1191-7.
17. Pinto ME, Tanuri A, Schechter M. Molecular and epidemiologic evidence for the discontinuous introduction of subtypes B and F into Rio de Janeiro, Brazil. J Acquired Immune Defic Syndr. In press 1998.
18. Louwagie J, Delwart EL, Mullins JI, McCutchan FE, Eddy G, Burke DS, et al. Genetic analysis of HIV-1 isolates from Brazil reveals presence of two distinct genetic subtypes. AIDS Res Hum Retroviruses 1994;10:561-7.
19. Morgado MG, Sabino EC, Shpaer EG, Bongertz V, Brigido L, Guimaraes MD, et al. V3 region polymorphisms in HIV-1 from Brazil: prevalence of subtype B strains divergent from North American/ European prototype and detection of subtype F. AIDS Res Hum Retroviruses 1994;10:569-76.
20. World Health Organization. HIV type 1 variation in World Health Organization-sponsored vaccine evaluation sites: genetic screening, sequence analysis, and preliminary biological characterization of selected viral strains. AIDS Res Hum Retroviruses 1994;10:1327-43.
21. Sabino EC, Diaz RS, Brigido LF, Learn GH, Mullins JI, Reingold AL, et al. Distribution of HIV-1 subtypes seen in an AIDS clinic in Sao Paulo City, Brazil. AIDS 1996;10:1579-84.
22. Sabino EC, Shpaer EG, Morgado MG, Korber BT, Diaz RS, Bongertz V, et al. Identification of human immunodeficiency virus type 1 envelope genes recombinant between subtypes B and F in two epidemiologically linked individuals from Brazil. J Virol 1994;68:6340-6.
23. Schechter M, Zajdenverg R, Machado LL, Pinto ME, Lima LA, Perez MA. Predicting CD4 counts in HIV-infected Brazilian individuals: a model based on the World Health Organization staging system. J Acquired Immune Defic Syndr 1994;7:163-8.
24. Zhu T, Wang N, Carr A, Wolinsky S, Ho D. Evidence for coinfection by multiple strains of human immunodeficiency virus type 1 subtype B in an acute seroconvertor. J Virol 1995;69:1324-7.
25. Delwart EL, Shpaer EG, Louwagie J, McCutchan FE, Grez M, Rubsamen-Waigmann H, et al. Genetic relationships determined by a DNA heteroduplex mobility assay: analysis of HIV-1 env genes. Science 1993;262:1257-61.
26. Ou CY, Ciesielski CA, Myers G, Bandea CI, Luo CC, Korber BT, et al. Molecular epidemiology of HIV transmission in a dental practice. Science 1992;256:1165-71.
27. Higgins DG, Sharp PM. Fast and sensitive multiple sequence alignments on a microcomputer. Comput Appl Biosci 1989;5:151-3
28. Maidak BL, Larsen N, McCaughey MJ, Overbeek R, Olsen GJ, Fogel K, et al. The Ribosomal Database Project. Nucleic Acids Res 1994;22:3485-7.
29. Felsenstein J. PHYLIP-phylogeny interference package (version 3.2). Cladistics 1989;5:164-6.
30. Meyerhans A, Vartanian JP, Wain-Hobson S. DNA recombination during PCR. Nucleic Acids Res 1990;18:1687-91.
31. Simmonds P, Balfe P, Peutherer JF, Ludlam CA, Bishop JO, Brown AJ. Human immunodeficiency virus-infected individuals contain provirus in small numbers of peripheral mononuclear cells and at low copy numbers. J Virol 1990;64:864-72.
32. World Health Organization. Acquired immunodeficiency syndrome (AIDS): interim proposal for a WHO staging system for HIV infection and disease. Wkly Epidemiol Rec 1990;65:221-8.
33. Cornelissen M, Kampinga G, Zorgdrager F, Goudsmit J, and the UNAIDS Network for HIV Isolation and Characterization. Human immunodeficiency virus type 1 subtypes defined by env show high frequency of recombinant gag genes. J Virol 1996;70:8209-12.
34. Tanuri A, Swanson P, Devare S, Berro OJ, Savedra A, Costa LJ, et al. HIV-1 subtypes among blood donors from Rio de Janeiro, Brazil. J Acquired Immune Defic Syndr. In press 1998.
